# Abdominal subcutaneous and visceral adipocyte size, lipolysis and inflammation relate to insulin resistance in male obese humans

**DOI:** 10.1038/s41598-018-22962-x

**Published:** 2018-03-16

**Authors:** K. Verboven, K. Wouters, K. Gaens, D. Hansen, M. Bijnen, S. Wetzels, C. D. Stehouwer, G. H. Goossens, C. G. Schalkwijk, E. E. Blaak, J. W. Jocken

**Affiliations:** 1 Department of Human Biology, NUTRIM School of Nutrition and Translational Research in Metabolism, Maastricht University Medical Centre+, Maastricht, The Netherlands; 2 Rehabilitation Research Center, BIOMED Biomedical Research Institute, Faculty of Medicine and Life Sciences, Hasselt University, Diepenbeek, Belgium; 3 Department of Internal Medicine, Cardiovascular Research Institute Maastricht (CARIM), Maastricht University Medical Centre+, Maastricht, The Netherlands; 4 Heart Centre Hasselt, Jessa Hospital, Hasselt, Belgium

## Abstract

Obesity is associated with a disturbed adipose tissue (AT) function characterized by adipocyte hypertrophy, an impaired lipolysis and pro-inflammatory phenotype, which contributes to insulin resistance (IR). We investigated whether AT phenotype in different AT depots of obese individuals with and without type 2 diabetes mellitus (T2DM) is associated with whole-body IR. Subcutaneous (SC) and visceral (V) AT biopsies from 18 lean, 17 obese and 8 obese T2DM men were collected. AT phenotype was characterized by *ex vivo* measurement of basal and stimulated lipolysis (mature adipocytes), adipocyte size distribution (AT tissue sections) and AT immune cells (flow cytometry). In VAT, mean adipocyte size, CD45^+^ leukocytes and M1 macrophages were significantly increased in both obese groups compared to lean individuals. In SCAT, despite adipocyte hypertrophy, no significant differences in immune cell populations between groups were found. In SCAT, multiple linear regression analysis showed that none of the AT phenotype markers independently contributed to HOMA-IR while in VAT, mean adipocyte size was significantly related to HOMA-IR. In conclusion, beside adipocyte hypertrophy in VAT, M1 macrophage- or B-cell-mediated inflammation, may contribute to IR, while inflammation in hypertrophic SCAT does not seem to play a major role in IR.

## Introduction

During the development of obesity, adipose tissue (AT) expansion frequently results in adipocyte hypertrophy (*i.e*. enlargement of the adipocyte), which is a known stressor for adipocytes^1^. Increases in AT mass and adipocyte volume result in a broad range of metabolic repercussions including, amongst others, a decreased insulin-mediated suppression^2,3^ and attenuated catecholamine or atrial natriuretic peptide (ANP)-mediated stimulation of AT lipolysis^4–7^. Especially in the postprandial state, this altered AT function is characterized by an impaired lipid buffering capacity^8^. Subsequently, this may lead to systemic lipid overflow and ectopic lipid accumulation in several insulin sensitive peripheral tissues like skeletal muscle, liver, pancreas, the heart and kidneys, which relates to the development of peripheral and systemic insulin resistance (IR)^9^.

Different immune cells are activated within hypertrophic AT (in case of resident immune cells) or attracted toward necrotic/apoptotic hypertrophic adipocytes, especially in the visceral AT (VAT), mainly based on rodent studies^10,11^. This VAT inflammation was proposed to be the strongest correlate of IR in human obesity^12,13^. In this regard, AT macrophages have gained much attention as important mediators of AT inflammation. In human obesity, AT macrophages display profound pro-inflammatory (M1) characteristics^14,15^ and are thereby thought to be the major source of pro-inflammatory cytokines and chemokines. This in turn may affect local AT lipolysis and further impede adipocyte function^16^ and reduce insulin sensitivity^17–19^. However, recent evidence indicates the presence of other innate and adaptive immune cells in the AT (reviewed by Mraz *et al*.)^20^. The cross-talk and dynamics of immune cells initiating and orchestrating AT inflammation and an impaired lipid metabolism in different AT depots are incompletely understood in the obese non-diabetic or type 2 diabetic state.

It is still an ongoing debate whether an impaired AT lipolysis or inflammation, or an interaction between both, is a cause or rather a consequence of IR in the obese state. Animal models propose a causative role of AT inflammation in the pathogenesis of IR^21^. In contrast, recent human studies indicated the development of hyperinsulinemia and dyslipidemia and (peripheral) IR upon overfeeding without affecting the inflammatory phenotype of the SCAT, suggesting that AT inflammation is not necessary to evoke peripheral IR in humans^22,23^. Of interest, healthy AT expansion and remodeling implies a certain degree of AT inflammation contrasting the general belief that inflammation negatively affects metabolism^24^.

Therefore, gaining better insights into the association between AT lipolysis, inflammation and IR is imperative to improve future development of therapeutic strategies to ameliorate the adverse metabolic consequences of obesity. In the present study, we studied subcutaneous and visceral AT morphology (adipocyte size), *ex vivo* lipolysis and immune cell populations (flow cytometry) in relation to whole-body IR in obese non-diabetic and type 2 diabetic (T2DM) men, compared with age-matched lean men.

## Methods

### Subjects

For this study, lean and obese age-matched male individuals who were scheduled to undergo laparoscopic abdominal (inguinal hernia or gallbladder removal) or bariatric surgery were recruited, as described previously in more detail^7^. The lean control group consisted of 18 male individuals. The obese group was composed of 25 male individuals, including 17 individuals without type 2 diabetes and 8 individuals with type 2 diabetes. Presence of type 2 diabetes was based on known clinical diagnosis (on average 2.5 years of diagnosis, ranging from newly diagnosed to 6 years). Obese diabetic individuals had glycated haemoglobin (HbA1c) levels ≥6.5% (45 mmol/mol) or were on glucose lowering medication. Major exclusion criteria were the use of exogenous insulin, presence or history of heart, lung or kidney disease and/or presence of endocrine anomalies. The study protocol was approved by the Medical Ethical Committee Jessa hospital, Hasselt, and Hasselt University, Belgium, in accordance with the Declaration of Helsinki (2008), and all individuals gave their written informed consent before participating in the study.

### Anthropometric measurements and blood sampling

Body weight, height, waist/hip circumference and blood pressure were determined at the morning of surgery. Fat and lean body mass were estimated by bio-electrical impedance analyses (Bodystat 1500; Bodystat Ltd., Isle of Man, UK). Fasting venous blood samples were collected after an overnight fast for measurement of plasma glucose, serum insulin and HbA1_C_. Insulin sensitivity was assessed by the homeostatic model assessment index for insulin resistance (HOMA-IR), calculated from fasting glucose and insulin, according to the formula: fasting insulin (mU/l)*fasting glucose (mmol/l)/22.5^25^. Plasma glucose concentration was measured by the glucose oxidase method using an AU2700 analyser (Beckman Coulter, Brea, CA, USA). Serum insulin concentration was assessed by immunoassay (ADVIA Centaur Insulin IRI; Siemens Medical Solutions Diagnostics, Tarrytown, NY, USA). HbA1_C_ was assessed by high performance liquid chromatography using a HA-8160 Hi-Auto A1C analyser (Menarini, Zaventem, Belgium).

### Abdominal subcutaneous and visceral adipose tissue biopsies

After an overnight fast, adipose tissue biopsies were taken from the periumbilical subcutaneous adipose tissue depot (SCAT) and the distal portion of the omentum majus (VAT). The tissue samples were immediately placed in saline and transported on ice to the laboratory for further processing. One portion of fresh AT was used for histological sections whereas another portion was used for isolation of the stromal vascular fraction (SVF) and mature adipocytes for lipolysis measurements.

### Adipocyte size

A small part of the AT samples was fixed overnight in 4% paraformaldehyde and embedded in paraffin. Histological sections (8 µm) were cut, mounted on microscope glass slides and dried overnight in an incubator (37 °C). Haematoxylin and eosin staining was used. Digital images were captured using a Leica DFC320 digital camera (Leica DM3000 microscope, Leica, Rijswijk, The Netherlands) at 20× magnification. Adipocyte size and distribution was performed in a blinded fashion (coefficient of variation <5%) using computerized morphometric analysis (Leica QWin V3, Cambridge, UK) of individual adipocytes (at least 400 adipocytes per sample), as described previously^26^.

### Adipocyte isolation, stromal vascular preparation and lipolysis measurement

Mature adipocytes and stromal vascular cells were obtained, as described previously^7^. Briefly, after collagenase digestion of AT fragments in Dulbecco’s modified Eagle’s medium (DMEM)-Ham’s F12, the resulting suspension was filtered and mature adipocytes were diluted in DMEM-Ham’s F12 supplemented with 3% BSA for lipolysis assays and incubated with isoprenaline (ISO, a non-selective β-adrenergic agonist; 10^−6^ M) or human ANP (Bachem) (10^−4^ M) for 3 h at 37 °C. Following incubation, glycerol concentration in the medium (lipolysis index) was determined using the EnzyChromeTM Adipolysis assay kit (Gentaur) and expressed per cell number or as maximal responsiveness to adrenergic or ANP stimulation (compared with baseline).

### Flow cytometry

Multicolour flow cytometry measurements were performed and analysed as described previously^27^. Briefly, isolated SVF cells, which were obtained following collagenase digestion and lysis of red blood cells, were stained for flow cytometry using two antibody cocktails. Cocktail 1 included CD45-PE-Cy7 (BD 557748), CD3-fitc (BD 561807), CD19-fitc (BD 555412), CD56-fitc (BD 562794), CD66b-fitc (BD 555724), CD11b-BV421 (Biologend 301324), and CD11c-APC-Cy7 (Biolegend 337218). Cocktail 2 included CD45-PE-Cy7 (BD 557748), CD3-V500 (BD 561416), CD4-PerCP (Biolegend 300528), CD8 APC-H7 (BD 641400), CD19-BV421 (Biolegend 302234), and CD56-APC (Biolegend 318310). Samples were measured with a FACS-Canto II (BD Biosciences). Results were analysed with FACSdiva (BD Biosciences) and FlowJo software. Since weight of the AT samples was unavailable, data are expressed as percentage of cells relative to total number of cells (based on forward and side scatter plot).

### Statistical analyses

All data are presented as mean ± S.E.M. Shapiro-Wilk tests were used to examine normality and variables with a skewed distribution were ln-transformed prior to analysis. Clinical characteristics with a skewed distribution were reported as median (interquartile range). Group differences were analysed using one-way ANOVA. Post-hoc testing was performed using Bonferroni adjustment for multiple testing when one-way ANOVA showed a significant group difference. Correlation analysis was applied by Pearson’s correlation. Multiple linear regression analyses including age, total fat mass and the AT phenotype markers that were significantly correlated with HOMA-IR were used to investigate whether AT morphology, lipolysis and inflammation were independently related to whole-body IR (dependent variable, expressed as HOMA-IR). These analyses were performed for both AT depots separately. Statistical significance was set at *p* < 0.05 (two-tailed). Analyses were performed using SPSS 22.0 for Windows (SPSS).

## Results

### Subjects’ characteristics

By design, no differences in age between lean individuals and both obese groups were observed. As expected, lean individuals’ body composition and metabolic parameters, including insulin sensitivity (expressed as HOMA-IR) and glycaemic control (indicated by fasting plasma glucose and HbA1c levels) were significantly different compared to the obese groups (Table 1). BMI, body fat percentage and body fat distribution was not significantly different between both obese groups, while blood pressure measures were comparable between groups. Fasting plasma glucose levels, whole-body IR (HOMA-IR) and glycated haemoglobin (HbA1_c_) were significantly elevated in the obese T2DM individuals as compared to obese individuals (Table 1). Details with respect to medication use in both obese groups are summarized in Supplementary Table S1.

**Table 1:** Characteristics of obese, obese diabetic individuals and healthy lean controls.

Variable	Lean individuals	Obese individuals	Obese diabetic individuals
*n*	18	17	8
Age, years	52 (48–57)	48 (45–54)	52 (48–56)
BMI, kg/m²	23.8 (22.7–25.0)	37.1 (35.4–38.7)^c^	36.7 (34.8–39.1)^c^
Body fat, %	22.1 (20.2–27.7)	35.1 (32.9–39.3)^c^	35.7 (33.8–37.9)^c^
Fat mass, kg	17.6 (14.8–20.6)	40.6 (39.5–48.9)^c^	41.0 (34.7–45.8)^c^
Waist circumference, cm	92.0 (87.5–94.5)	124.5 (123.2–129.2)^c^	123.5 (120.6–130.1)^c^
Hip circumference, cm	94.0 (89.8–97.2)	115.0 (112.0–118.0)^c^	116.7 (110.7–122.2)^c^
Waist-to-hip ratio	0.98 (0.95–0.99)	1.08 (1.06–1.10)^c^	1.07 (1.02–1.13)^c^
Systolic blood pressure, mmHg	127 (120–138)	140 (130–150)	147 (136–155)
Diastolic blood pressure, mmHg	80 (80–92)	80 (80–90)	82 (80–96)
Fasting plasma glucose, mmol/l	5.5 (5.2–5.9)	5.6 (5.2–6.3)	6.8 (5.9–8.7)^c,d^
Serum insulin, mU/l	7.2 (5.5–11.4)	19.0 (15.0–31.0)^c^	15.0 (12.0–19.0)
HbA1c, %	5.2 (5.1–5.5)	5.6 (5.4–5.7)	6.7 (6.2–7.7)^c,e^
HbA1c, mmol/mol	33 (31–40)	38 (32–54)^a^	50 (42–67)^c,e^
HOMA-IR	1.7 (1.2–2.8)	4.7 (3.3–7.7)^c^	4.7 (3.5–6.0)^a^
Subcutaneous adipocyte diameter, µm*	63.4 (59.0–72.1)	75.6 (72.8–85.6)^c^	73.8 (70.9–77.4)
Visceral adipocyte diameter, µm	60.3 (50.8–69.7)	80.6 (73.4–86.1)^c^	78.1 (76.0–87.9)^c^

Data are median (interquartile range). *Data from 14 lean, 16 obese and 7 obese diabetic individuals. Significantly different from lean group (^a^*p < *0.05; ^b^*p* < 0.01; ^c^*p* < 0.001). Significantly different from obese group (^d^p < 0.01; ^e^p < 0.001).

### SCAT and VAT morphology

**Figure 1:**
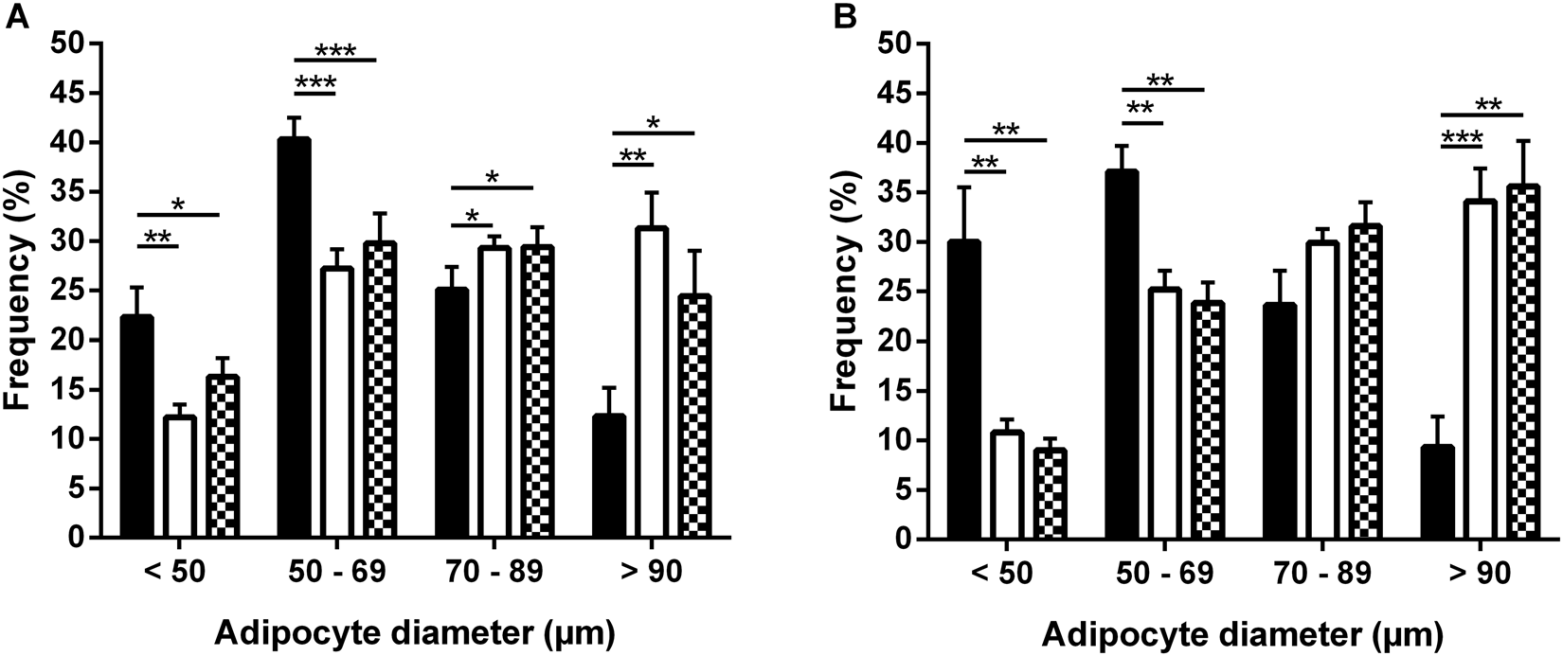
Adipocyte size distribution. The frequency of small (<50 µm), medium (50–69 µm), large (70–89 µm) and very large (>90 µm) adipocytes was determined in SCAT (**A**) and VAT (**B**) of lean (black bars), obese (white bars) and obese diabetic (white squared bars) individuals. Obese groups clearly had higher proportions of large and very large adipocytes in combination with smaller proportions of small and medium size adipocytes, resulting in a higher mean adipocytes size compared to lean individuals both in SCAT (*p* < 0.001 for obese individuals and *p* < 0.01 for obese diabetic individuals) and VAT (*p* < 0.001 for both obese groups) Data are means ± SEM. *p < 0.05, **p < 0.01, ***p < 0.001.

Mean adipocyte size was clearly elevated in both obese groups as compared to the lean group in both SCAT (*p* < 0.001 for obese individuals) and VAT (*p* < 0.001 for both obese and obese T2DM individuals) (Table 1). However, there was no significant difference in mean adipocyte size between the different AT depots within obese groups (Table 1). The difference in mean adipocyte size between lean and obese individuals was attributed by a shift toward a significantly higher proportion of large (70–89 µm) and very large (>90 µm) adipocytes, and a lower proportion of small (<50 µm) and medium size (50–69 µm) adipocytes in the obese individuals, in both the SCAT (Fig. 1A) and VAT (Fig. 1B). Detailed lipolysis data for the individuals in Table 1 have been reported previously^7^. Briefly, we observed an increased basal lipolysis in the SCAT and VAT of obese and obese diabetic individuals and an attenuated maximal ANP- and ISO-induced lipolysis in the SCAT of obese diabetic men as compared to lean individuals (with intermediate values for obese individuals).

### Elevated inflammatory cell populations in the SVF of obese VAT

In SCAT, no significant differences were found in immune cell populations, expressed as percentage of total cells, between groups (Table 2). In contrast, the VAT of both obese groups showed a clear increase in proportion of leukocytes (CD45^+^) numbers, expressed as percentage of total cells, compared to lean controls (*p* < 0.001 for obese individuals and *p* < 0.01 for obese T2DM individuals). However, no significant differences were found between groups for the proportion of B-cells (CD19^+^), T-cell subsets (T-helper cells or cytotoxic T-cells) or NK cells (CD56^+^) (Table 2). For VAT macrophages, we observed an increased proportion of M1-macrophages (CD11c^+^; expressed as percentage of total cells) in obese individuals (*p* < 0.01) compared to lean controls. A trend toward a higher proportion of CD11c^−^ M2 macrophages in obese VAT was observed (*p*_group_ = 0.088) (Table 2). No group differences were observed when representing cell subsets as a fraction of total parent cells (either as % of CD45^+^ lymphocytes or as % of macrophages), thereby reflecting qualitative characteristics (Supplementary Table S2).

**Table 2:** Frequency of cell populations in subcutaneous and visceral SVF determined by flow cytometry.

Variable	Lean individuals	Obese individuals	Obese diabetic individuals	P_group_
*n*	18	17	8	
*Subcutaneous SVF*				
Total CD45+ leukocytes	41.3 ± 4.2 (4.3–70.4)	43.0 ± 3.9 (18.0–74.0)	38.8 ± 5.5 (23.5–74.0)	0.847
B cells	0.79 ± 0.16 (0.10–2.44)	1.26 ± 0.32 (0.14–5.20)	0.98 ± 0.40 (0.17–3.00)	0.291
T cells	21.9 ± 2.7 (1.8–48.9)	25.1 ± 1.8 (7.3–35.7)	24.1 ± 3.4 (16.3–42.3)	0.643
CD4+ T helper cells	12.0 ± 2.0 (0.8–33.3)	11.9 ± 1.0 (3.3–19.9)	13.2 ± 2.8 (7.1–28.1)	0.917
CD8+ cytotoxic T cells	6.5 ± 0.7 (0.6–10.8)	8.0 ± 0.7 (2.5–11.5)	7.4 ± 1.2 (3.8–13.1)	0.366
CD4+/CD8+ ratio	1.8 ± 0.2 (0.4–3.7)	1.5 ± 0.1 (0.8–2.9)	1.9 ± 0.3 (0.5–2.9)	0.818
NK cells	2.7 ± 0.5 (0.1–8.1)	3.7 ± 0.3 (1.8–6.1)	3.8 ± 1.1 (0.5–7.8)	0.298
M1 macrophages	6.6 ± 1.2 (0.2–16.1)	10.1 ± 1.3 (0.2–19.6)	6.4 ± 1.2 (2.2–12.0)	0.098
M2 macrophages	5.1 ± 1.0 (0.4–15.5)	5.5 ± 1.4 (0.6–22.8)	3.4 ± 0.8 (0.3–8.3)	0.810
M1/M2 ratio	2.1 ± 0.6 (0.4–11.5)	3.1 ± 0.6 (0.3–8.6)	2.8 ± 0.8 (0.6–8.2)	0.283
*Visceral SVF*				
Total CD45+ leukocytes	34.9 ± 3.5 (5.6–54.3)	57.4 ± 3.2 (33.0–93.8) ^c^	54.0 ± 2.6 (41.6–65.7) ^b^	**<0.001**
B cells	1.7 ± 0.5 (0.1–7.5)	4.0 ± 1.7 (0.0–30.4)	1.3 ± 0.1 (0.7–2.0)	0.488
T cells	28.7 ± 3.3 (3.3–59.3)	34.4 ± 3.4 (15.1–56.2)	37.9 ± 4.5 (12.7–51.4)	0.260
CD4+ T helper cells	15.3 ± 2.1 (1.7–31.8)	16.8 ± 1.8 (0.3–28.6)	18.6 ± 2.7 (6.6–28.9)	0.619
CD8+ cytotoxic T cells	10.1 ± 1.2 (0.9–18.3)	13.4 ± 1.9 (3.3–25.0)	15.6 ± 2.5 (5.2–26.4)	0.122
CD4+/CD8+ ratio	1.7 ± 0.2 (0.7–5.2)	1.7 ± 0.3 (0.0–5.8)	1.3 ± 0.2 (0.5–2.2)	0.690
NK cells	3.0 ± 0.4 (0.0–7.4)	3.4 ± 0.5 (0.2–8.9)	4.1 ± 0.9 (1.0–8.6)	0.593
M1 macrophages	2.9 ± 0.5 (0.1–9.0)	6.3 ± 0.9 (1.3–12.7) ^b^	4.2 ± 0.8 (1.2–8.7)	**0.010**
M2 macrophages	3.5 ± 0.5 (0.1–8.5)	7.4 ± 1.4 (0.6–24.1)	6.2 ± 1.6 (0.9–17.0)	0.088
M1/M2 ratio	0.9 ± 0.1 (0.2–2.2)	1.4 ± 0.3 (0.1–5.3)	1.2 ± 0.4 (0.2–4.3)	0.628

Data are mean ± S.E.M (range). Cell frequencies are expressed as % of total cells; NK, natural killer; CD, cluster of differentiation; SVF, stromal vascular fraction. Significantly different from lean group (^a^*p* < 0.05; ^b^*p* < 0.01; ^c^*p* < 0.001).

### Determinants of whole-body insulin resistance

First, we investigated whether AT functional variables (adipocyte size, basal lipolysis and maximal lipolytic responsiveness to ISO or ANP) and AT immunophenotype were associated with whole-body IR, estimated using HOMA-IR (Table 3). In SCAT, basal lipolysis (expressed per cell number; *p* = 0.002) and mean adipocyte size (*p* = 0.004) were positively correlated with HOMA-IR. Furthermore, SCAT CD3^+^ T-cells (*p* = 0.018), CD8^+^ cytotoxic T-cells (*p* = 0.001) and CD56^+^ NK-cells (*p* = 0.005) correlated positively with HOMA-IR (Table 3). In VAT, mean adipocyte size correlated positively (*p* < 0.001) with HOMA-IR. Moreover, VAT CD45^+^ leukocytes (*p* = 0.001) and CD19^+^ B-cells (*p* = 0.034) were positively correlated with HOMA-IR, while VAT CD8^+^ cytotoxic T-cells tended to show a positive association with HOMA-IR (r = 0.29, *p* = 0.069) (Table 3). Of interest, AT functional variables showed some correlations with AT immunophenotype in the SCAT as well as in the VAT, respectively (Supplementary Table S3).

**Table 3:** Pearson correlation coefficients between AT lipolysis, AT size and AT immunophenotype of subcutaneous and visceral AT depots and whole-body IR.

*Subcutaneous AT*	R	p value	*Visceral AT*	R	p value
Basal lipolysis, µmol glycerol (3 h) per 10^6 cells	**0.525**	**0.002**	Basal lipolysis, µmol glycerol (3 h) per 10^6 cells	0.118	0.514
Isoprenaline responsiveness, fold change to basal	−0.242	0.175	Isoprenaline responsiveness, fold change to basal	−0.290	0.102
ANP responsiveness, fold change to basal	−0.237	0.184	ANP responsiveness, fold change to basal	−0.167	0.361
Adipocyte diameter, µm	**0.472**	**0.004**	Adipocyte diameter, µm	**0.652**	**<0.001**
Total CD45+ leukocytes, % of total cells	0.192	0.234	Total CD45+ leukocytes, % of total cells	**0.514**	**0.001**
CD3+ T cells, % of total cells	**0.381**	**0.018**	CD3+ T cells, % of live cells	0.166	0.300
CD3+ CD4+ T-helper cells, % of total cells	0.204	0.219	CD3+ CD4+ T-helper cells, % of total cells	0.016	0.923
CD3+ CD8+ cytotoxic T cells, % of total cells	**0.499**	**0.001**	CD3+ CD8+ cytotoxic T cells, % of total cells	0.290	0.069
CD4+/CD8+ T cell ratio	−0.095	0.570	CD4+/CD8+ T cell ratio	−0.128	0.431
CD56+ NK cells, % of total cells	**0.442**	**0.005**	CD56+ NK cells, % of total cells	0.206	0.196
CD11c+ M1 macrophages, % of total cells	0.086	0.598	CD11c+ M1 macrophages, % of total cells	0.243	0.127
CD11c− M2 macrophages, % of total cells	−0.105	0.521	CD11c- M2 macrophages, % of total cells	0.195	0.221
M1/M2 ratio	0.060	0.715	M1/M2 ratio	0.078	0.626
CD19+ B lymphocytes, % of total cells	0.047	0.778	CD19+ B lymphocytes, % of total cells	**0.332**	**0.034**

ANP, atrial natriuretic peptide; AT, adipose tissue; NK, natural killer; CD, cluster of differentiation.

Subsequently, multiple linear regression analyses including age, total fat mass and the AT phenotype markers that were significantly correlated with HOMA-IR (indicated in Table 3) were used to investigate whether AT morphology, lipolysis and inflammation were independently related to whole-body IR (dependent variable, expressed as HOMA-IR). In SCAT, none of the AT phenotype markers were significantly associated to HOMA-IR (Table 4). In VAT, adipocyte size (β = 0.599, p = 0.002) was significantly associated to HOMA-IR, explaining about 17% the variance in HOMA-IR (*p*_model_ < 0.001) (Table 4).

**Table 4:** Associations between whole-body insulin sensitivity and depot-specific adipose tissue characteristics.

SCAT model*	Variable	β (95%-CI)	p-value
	basal lipolysis (corrected for cell count)	0.108 (0.000; 0.000)	0.527
	adipocyte diameter	0.131 (−0.022; 0.044)	0.487
	CD3+ T cells	−0.117 (−0.051; 0.031)	0.602
	CD3+ CD8+ cytotoxic T cells	0.313 (−0.038; 0.218)	0.159
	CD56+ NK cells	0.261 (−0.049; 0.269)	0.166
VAT model*	**Variable**	**β (95%-CI)**	**p-value**
	adipocyte diameter	0.599 (0.016; 0.062)	**0.002**
	total CD45+ leukocytes	−0.116 (−0.024; 0.012)	0.515
	CD19+ B lymphocytes	0.217 (−0.008; 0.085)	0.105

*Model adjusted for age and fat mass. Multiple linear regression analyses in lean (n = 18), obese (n = 17) and obese diabetic (n = 8) individuals. The beta of each predictor represents standardized beta along with its respective 95%-CI and p-value.

## Discussion

To the best of our knowledge, this is the first study reporting detailed data on the relationship between depot-specific AT phenotype (*i.e*. morphology, lipolysis and inflammation) and whole-body IR (HOMA-IR) in male lean, obese non-diabetic individuals and obese humans with type 2 diabetes. As reported previously^7^, obese individuals have an attenuated basal lipolysis in both SCAT and VAT, and a decreased maximal ANP- and ISO-mediated lipolytic responsiveness in SCAT, compared to age-matched lean individuals. Here we demonstrate that this altered AT phenotype was further characterized by adipocyte hypertrophy and an increased immune cell infiltration, especially in the VAT. Of interest, in the VAT adipocyte size was positively related to whole-body IR. In contrast, none of the subcutaneous AT functional measures or immunophenotype were independently related to whole-body IR.

The present findings indicate that adipocyte hypertrophy in the obese SCAT and VAT is a major determinant of whole-body IR, which is in line with previous studies^28–30^. In addition, VAT adipocyte hypertrophy was independently and positively associated (β = 0.599, p = 0.002) with whole-body IR, also after adjustment for age and total fat mass. Although adipocyte hypertrophy is the preferential expansion mechanism within the AT, in the chronic state of obesity SCAT adipogenesis is commonly impaired^31^, possibly resulting in lipid spill-over to other tissues (the VAT as well) and thus the development of metabolic complications^32^. The latter may explain our finding that adipocyte hypertrophy in VAT was an independent determinant of whole-body IR. Importantly, abdominal subcutaneous adipocyte hypertrophy may thus substantially contribute to lipid overflow, ectopic lipid deposition and expansion of VAT, which in turn results in worsening of whole-body IR.

The presence of a chronic pro-inflammatory AT microenvironment may limit adipocyte hypertrophy, by activating fibrotic signalling and impairing adipocyte functionality^33^, thereby contributing to metabolic alterations frequently observed in the obese state^34,35^. In addition to adipocyte hypertrophy, low-grade AT inflammation is essential for healthy AT expansion and remodelling, as some cells are implicated in the clearance of cell debris released by necrotic adipocytes^36^, act as a local buffer against increased lipid content^37^, and promote angiogenesis^38^. In this regard, our data show a significantly increased proportion of CD45^+^ leukocytes and CD11c^+^ M1 macrophages in the VAT of obese and obese diabetic humans. In contrast, an inflammatory phenotype was less pronounced in the SCAT of obese individuals compared to lean or obese T2DM individuals, where we only observed a trend toward a higher proportion of CD11c^+^ M1 (pro-inflammatory) macrophages in the obese SCAT (Table 2). Additionally, no immune-related markers in SCAT were significantly related to HOMA-IR, when adjusting for total fat mass and age. Indeed, the VAT is believed to have a more pronounced inflammatory phenotype than the SCAT^39^, which may possibly be ascribed to an increased production of inflammatory mediators by adipocytes themselves and by cells in the SVF^40^. Concerning the latter, one should be aware that SCAT is more important in quantitative terms, since SCAT accounts for approximately 80% of total AT mass. This may also explain why total fat mass rather than adipocyte morphology or the SCAT function was the strongest independent determinant of whole-body IR.

Therefore, in the VAT, the potential involvement of the elevated immune cells (*i.e*. CD45^+^ leukocytes and CD11c^+^ M1 macrophages) in the development of AT inflammation and IR in obese humans is likely, indicated by a strong positive correlation between these CD45^+^ leukocytes and HOMA-IR (r = 0.514; p = 0.001) and a tendency for a positive correlation between CD8^+^ cytotoxic T-cells and HOMA-IR (r = 0.290; p = 0.069) (Table 3). Notably, VAT CD19^+^ B (r = 0.332, p = 0.034) cells were positively correlated with HOMA-IR. B lymphocytes are known to be elevated in obesity and IR, at least in diet-induced obese mice^41^. In epididymal AT, by orchestrating T cell populations and their activity (*i.e*. production of pro-inflammatory cytokines) and the release of pathogenic antibodies, B cells can decrease glucose tolerance^41^. Recently, the pro-inflammatory features of B cells in a high-fat diet setting were confirmed in obese mice^42^. These data, together with the present findings, suggest that B cells exert pro-inflammatory effects in obese AT and may contribute to IR. However, the precise mechanisms via which these cells impair insulin sensitivity require further investigation, especially in human obesity.

Inflammatory cells may have a modulatory function in chronically increased lipolysis. Indeed, beside the presence of inflammation additional mechanisms that may be (in)directly related to inflammation may be of importance in worsening of IR in obesity, as previously suggested^43^. Of interest, Kosteli *et al*.^37^ suggested that AT macrophages may buffer excess intracellular fatty acids upon increased lipolysis during fasting. The chronic elevation and uptake of lipids by macrophages may alter their phenotype^44^, promoting the progression to an inflammatory (CD11c^+^) M1 polarisation and AT inflammation^45^. However, in the present study no association between SCAT or VAT immunophenotype and measures of lipolysis were observed, suggesting that adipocyte lipolysis does not activate inflammatory pathways in human AT macrophages, as shown recently^46^. In line, only in the VAT a correlation between M1 and M2 macrophages and adipocyte size was found (Supplementary Table S3), respectively, suggesting that different immunological processes or alternative stimuli for AT macrophages (*e.g*. concentration of saturated fatty acids or ratio of monounsaturated to saturated fatty acids) could be present between abdominal SCAT and VAT depots in humans. Moreover, altered AT oxygen tension may have a putative role in AT dysfunction in obese insulin resistant conditions, as recently reviewed^47^. However, further studies are warranted to obtain more insight into the triggers of AT dysfunction within different AT depots.

Future studies should include human mechanistic experiments to strengthen these findings by focussing on intra-AT relationships and to address causality in a larger sample size. Our study was also performed in male individuals and it is not known whether the outcomes can be extrapolated to females, since it is evident that there are sex-specific differences in fat metabolism and white AT function^48^. In addition, it needs to be mentioned that a significant proportion of the obese patients in the present study received several forms of glucose lowering or lipid lowering medication (Supplementary Table S1), which may impact the AT phenotype and have cofounded the present results^49,50^. Furthermore, the use of HOMA-IR as a surrogate markers to assess whole-body IR and bioimpedance measurements to determine body composition may be two limitations of this study. It would be of interest to relate AT functional characteristics to tissue-specific (*e.g*. muscle, AT or liver) IR, which need to be determined in future studies using for example a two-step hyperinsulinemic-euglycemic clamp combined with a glucose tracer.

In conclusion, this study is the first to characterize the SCAT and VAT phenotype in detail in male lean, obese and obese type 2 diabetic subjects. In VAT, adipocyte hypertrophy independently contributes to IR, possibly via pro-inflammatory M1 macrophage- and/or B cell-mediated inflammation. In SCAT, AT function or immunophenotype were only of minor importance with respect to whole-body IR. These alterations in AT mass and phenotypes likely impact cardiometabolic risk and may contribute to the development of type 2 diabetes.

## Electronic supplementary material

Supplemental tables
