# Supplementary material for: Abdominal subcutaneous and visceral adipocyte size, lipolysis and inflammation relate to insulin resistance in male obese humans
Source: Sci Rep. 2018 Mar 16;8:4677. doi: 10.1038/s41598-018-22962-x (PMC5856747; doi:10.1038/s41598-018-22962-x)
Supplement: Supplementary file 1 — Supplemental tables [file 41598_2018_22962_MOESM1_ESM.docx]

Abdominal subcutaneous and visceral adipocyte size, lipolysis and inflammation relate to insulin resistance in male obese humans

Verboven K^1,2^, Wouters K^3^, Gaens K^3^, Hansen D^2,4^, Bijnen M^3^, Wetzels S^3^,

Stehouwer CD^3^, Goossens GH^1^, Schalkwijk CG^3^, Blaak EE^1^, Jocken JW^1^

^1^ Department of Human Biology, NUTRIM School of Nutrition and Translational Research in Metabolism, Maastricht University Medical Centre^+^, Maastricht, The Netherlands
^2^ Rehabilitation Research Center, BIOMED Biomedical Research Institute, Faculty of Medicine and Life Sciences, Hasselt University, Diepenbeek, Belgium
^3^ Department of Internal Medicine, Cardiovascular Research Institute Maastricht (CARIM), Maastricht University Medical Centre^+^, Maastricht, The Netherlands
^4^ Heart Centre Hasselt, Jessa Hospital, Hasselt, Belgium

**Supplemental tables**

**Supplemental Table S1 –** Subjects’ medication use details

**Supplemental Table S2 -** Frequency of cell populations in subcutaneous and visceral SVF, percentage relative to leukocytes or to macrophages

**Supplemental table S3** - Pearson correlation coefficients between adipocyte size and lipolysis with immuno-phenotype in subcutaneous and visceral adipose tissue

| **Supplemental table S1**  Subjects' medication use details | | |
| --- | --- | --- |
|  | Obese individuals | Obese diabetic individuals |
| *Cardiovascular disease* |  |  |
| antithrombotic | 4/17 | 4/8 |
| beta-blocker | 3/17 | 3/8 |
| ACE-inhibitor | 4/17 | 4/8 |
| calcium antagonist | 3/17 | 1/8 |
|  |  |  |
| *Glycemic control* |  |  |
| glucose lowering drugs | - | 6/8 |
| exogenous insulin | - | 0/8 |
|  |  |  |
| lipid lowering drugs | 4/17 | 2/8 |
| others | 7/17 | 7/8 |

| **Supplemental Table S2** Frequency of cell populations in subcutaneous and visceral SVF, expressed as percentage relative to CD45^+^   leukocytes or to total macrophages | | | | | | | |
| --- | --- | --- | --- | --- | --- | --- | --- |
|  |  |  |  |  |  |  |  |
| Variable |  | Lean individuals |  | Obese individuals |  | Obese diabetic individuals | P_group_ |
| *n* |  | 18 |  | 17 |  | 8 |  |
| *Subcutaneous SVF* |  |  |  |  |  |  |  |
|  |  |  |  |  |  |  |  |
| B cells (% of CD45^+^) |  | 1.8 ± 0.2 (0.5-4.5) |  | 2.1 ± 0.4 (0.4-7.2) |  | 1.6 ± 0.5 (0.4-4.2) | 0.669 |
| T cells (% of CD45^+^) |  | 50.1 ± 2.3 (32.4-69.4) |  | 43.0 ± 3.1 (14.9-67.5) |  | 41.8 ± 4.7 (15.1-55.5) | 0.133 |
| CD4+ T helper cells (% of CD45^+^) |  | 25.9 ± 2.1 (10.1-42.6) |  | 20.3 ± 1.7 (1.8-29.1) |  | 22.5 ± 3.7 (8.2-39.2) | 0.172 |
| CD8+ cytotoxic T cells (% of CD45^+^) |  | 16.4 ± 1.6 (8.1-39.8) |  | 13.4 ± 1.5 (1.8-28.0) |  | 14.0 ± 2.1 (8.7-26.0) | 0.394 |
| NK cells (% of CD45^+^) |  | 6.3 ± 0.9 (0.7-17.8) |  | 7.1 ± 0.9 (2.4-20.0) |  | 6.7 ± 1.5 (1.1-11.6) | 0.842 |
| M1 macrophages (% of macrophages) |  | 54.4 ± 5.1 (12.8-93.8) |  | 62.4 ± 5.1 (5.2-83.9) |  | 55.0 ± 8.3 (20.5-85.5) | 0.527 |
| M2 macrophages (% of macrophages) |  | 45.5 ± 5.1 (6.1-87.1) |  | 37.5 ± 5.1 (16.0-94.7) |  | 44.9 ± 8.3 (14.4-79.4) | 0.527 |
|  |  |  |  |  |  |  |  |
| *Visceral SVF* |  |  |  |  |  |  |  |
|  |  |  |  |  |  |  |  |
| B cells (% of CD45^+^) |  | 3.6 ± 1.3 (0.3-23.0) |  | 5.2 ± 1.9 (0.0-34.2) |  | 2.2 ± 0.2 (1.0-2.9) | 0.526 |
| T cells (% of CD45^+^) |  | 58.7 ± 2.2 (43.4-77.7) |  | 51.5 ± 4.3 (17.0-83.0) |  | 59.4 ± 5.2 (28.2-69.8) | 0.263 |
| CD4+ T helper cells (% of CD45^+^) |  | 30.9 ± 2.0 (16.7-46.1) |  | 25.2 ± 2.7 (0.6-46.4) |  | 28.8 ± 3.2 (14.7-41.6) | 0.246 |
| CD8+ cytotoxic T cells (% of CD45^+^) |  | 20.9 ± 1.6 (8.7-33.1) |  | 18.6 ± 2.5 (0.0-34.7) |  | 24.6 ± 3.2 (11.6-39.9) | 0.306 |
| NK cells (% of CD45^+^) |  | 6.6 ± 1.0 (0.0-16.1) |  | 5.4 ± 0.9 (0.3-15.5) |  | 7.4 ± 2.1 (1.6-19.0) | 0.549 |
| M1 macrophages (% of macrophages) |  | 45.3 ± 3.8 (19.4-79.4) |  | 50.3 ± 5.1 (11.1-84.1) |  | 52.0 ± 7.2 (23.5-81.2) | 0.630 |
| M2 macrophages (% of macrophages) |  | 54.6 ± 3.8 (20.5-80.5) |  | 49.6 ± 5.1 (15.8-88.8) |  | 47.9 ± 7.2 (18.7-76.4) | 0.630 |
|  |  |  |  |  |  |  |  |
| Data are mean ± S.E.M (range). Cell frequencies are expressed as % of CD45^+^ leukocytes or % of total macrophages (as indicated); NK, natural killer; CD, cluster of differentiation; SVF, stromal vascular fraction. | | | | | | | |

| **Supplemental table S3**  Pearson correlation coefficients between adipocyte size and lipolysis with immuno-phenotype in subcutaneous and   visceral adipose tissue | | | | | | |
| --- | --- | --- | --- | --- | --- | --- |
| ***Subcutaneous adipocyte size (µm)*** | **R** | **p value** |  | ***Visceral adipocyte size (µm)*** | **R** | **p value** |
| Total CD45+ leukocytes, % of total cells | 0.192 | 0.261 |  | Total CD45+ leukocytes, % of total cells | **0.672** | **< 0.001** |
| CD3+ T cells, % of total cells | 0.247 | 0.160 |  | CD3+ T cells, % of total cells | 0.259 | 0.102 |
| CD3+CD4+ T-helper cells, % of total cells | 0.100 | 0.573 |  | CD3+CD4+ T-helper cells, % of total cells | 0.219 | 0.168 |
| CD3+CD8+ cytotoxic T cells, % of total cells | **0.357** | **0.038** |  | CD3+CD8+ cytotoxic T cells, % of total cells | 0.218 | 0.177 |
| CD4+/CD8+ T cell ratio | -0.178 | 0.314 |  | CD4+/CD8+ T cell ratio | 0.020 | 0.900 |
| CD56+ NK cells, % of total cells | 0.119 | 0.502 |  | CD56+ NK cells, % of total cells | 0.106 | 0.510 |
| CD11+ M1 macrophages, % of total cells | 0.315 | 0.062 |  | CD11+ M1 macrophages, % of total cells | **0.520** | **< 0.001** |
| CD11- M2 macrophages, % of total cells | 0.183 | 0.286 |  | CD11- M2 macrophages, % of total cells | **0.407** | **0.008** |
| M1/M2 ratio | -0.028 | 0.871 |  | M1/M2 ratio | 0.112 | 0.487 |
| CD19+ B lymphocytes, % of total cells | 0.000 | 0.999 |  | CD19+ B lymphocytes, % of total cells | 0.190 | 0.233 |
|  | **R** | **p value** |  |  | **R** | **p value** |
| ***Subcutaneous basal lipolysis (per cell)*** |  |  |  | ***Visceral basal lipolysis (per cell)*** |  |  |
| Total CD45+ leukocytes, % of total cells | -0.158 | 0.372 |  | Total CD45+ leukocytes, % of total cells | **0.339** | **0.047** |
| CD3+ T cells, % of total cells | 0.067 | 0.709 |  | CD3+ T cells, % of total cells | 0.127 | 0.467 |
| CD3+CD4+ T-helper cells, % of total cells | 0.027 | 0.880 |  | CD3+CD4+ T-helper cells, % of total cells | 0.196 | 0.258 |
| CD3+CD8+ cytotoxic T cells, % of total cells | -0.086 | 0.633 |  | CD3+CD8+ cytotoxic T cells, % of total cells | 0.116 | 0.512 |
| CD4+/CD8+ T cell ratio | 0.168 | 0.350 |  | CD4+/CD8+ T cell ratio | 0.125 | 0.480 |
| CD56+ NK cells, % of total cells | 0.210 | 0.240 |  | CD56+ NK cells, % of total cells | 0.237 | 0.170 |
| CD11+ M1 macrophages, % of total cells | -0.005 | 0.979 |  | CD11+ M1 macrophages, % of total cells | 0.242 | 0.160 |
| CD11- M2 macrophages, % of total cells | -0.082 | 0.647 |  | CD11- M2 macrophages, % of total cells | 0.129 | 0.462 |
| M1/M2 ratio | -0.045 | 0.803 |  | M1/M2 ratio | -0.027 | 0.877 |
| CD19+ B lymphocytes, % of total cells | 0.104 | 0.565 |  | CD19+ B lymphocytes, % of total cells | -0.105 | 0.547 |
|  |  |  |  |  |  |  |
| Pearson correlations coefficients with adipocyte size or basal lipolysis (expressed per amount of cells) as dependent variables per depot. NK, natural killer; CD, cluster of differentiation | | | | | | |
